# Apropos: An update on the incidence of dengue gaining strength in Saudi Arabia and current control approaches for its vector mosquito

**DOI:** 10.1186/1756-3305-7-322

**Published:** 2014-07-11

**Authors:** Subhash C Arya, Nirmala Agarwal

**Affiliations:** 1 Sant Parmanand Hospital 18, Alipore Road, Delhi 110054, India

## To the Editor

We compliment the team of investigators from Saudi Arabia and Malaysia for their meticulous update on the incidence of dengue in Saudi Arabia as well as the population control of the vector mosquito, *Aedes aegypti*[1]. In our opinion, it is essential to disseminate information about the basics of the vector biology to control episodes of dengue virus infection among natives and travelers in Saudi Arabia.

The vector responsible for dengue virus in Saudi Arabia [1], *Aedes aegypti* is a day biter, but will bite at night if there is sufficient artificial lighting. *Aedes aegypti* usually bites at the ankles of humans [2]. *Aedes albopictus* is a very aggressive daytime biter, with peaks during early morning or late afternoon. Both are container-inhabiting species that lay eggs in clean water-containing receptacles in and around the home environment.

During the initial phase of illness, a patient is viremic with circulating viral RNA and non-structural protein NS1 antigen. Later the viral RNA and NS1 antigen disappear with a coincident appearance of antibody, although NS1 antigen may be detectable in a few patients for a small number of days after defervescence [3]. If any of the hospitalized cases have been bitten by a mosquito, circulating dengue virus could be picked up by the mosquitoes with its amplification in the mosquito tissues, leading to secondary dengue transmission.

Generally, anti-mosquito measures are practiced at night when repellents, insecticides, or mosquito nets are used. Even after nocturnal caretaking, one could still be exposed to bites from the Aedes group of mosquito.

The natives and expatriates to Saudi Arabia [1] should be advised to cover their arms and legs during day-time as well as in the evenings if they are out of their homes. It has been noticed that hospitals where dengue patients are being treated become the focal point for the spread of disease among hospital staff and neighboring populations [4]. Therefore, necessary precautions, such as the use of insecticide-impregnated bed nets, should be used even during the day time to avoid such situations. The basics of the mosquito biology like its breeding in fresh water, preference for in and around home environment should be disseminated among the scientific community as well as the Saudi natives and expatriates. The population at risk should be made aware about the mode of dengue transmission through audio-visual presentations in educational institutions, supermarkets as well as other public congregations.

As a final suggestion, fashion designers in Saudi Arabia should be urged to create formal and informal clothing for both sexes which would protect against mosquito bites and that are also suited to local customs in each region. Although Saudi males wear long white clothing and females would cover themselves wearing black clothing while outdoors, such dress would not be worn inside the house when informal dress would be used as a rule rather than an exception. Consequently, informal attire for indoor usage during day-time would protect individuals from the bites of *Aedes* group of mosquitoes. Prominent public personalities like film stars and sports personalities should wear such clothes during their appearances in public. School children should avoid use of shorts or skirts in the school. Local governments may offer subsidy to ensure reduced cost of such clothes

## Competing interests

The authors declare that they have no competing interests.

## Authors’ contributions

Both authors contributed to, read and approved the final version of the manuscript.
